# Apolipoprotein E4 (1–272) fragment is associated with mitochondrial proteins and affects mitochondrial function in neuronal cells

**DOI:** 10.1186/1750-1326-4-35

**Published:** 2009-08-20

**Authors:** Toshiyuki Nakamura, Atsushi Watanabe, Takahiro Fujino, Takashi Hosono, Makoto Michikawa

**Affiliations:** 1 Department of Alzheimer's Disease Research, National Institute for Longevity Sciences, National Center for Geriatrics and Gerontology, 36-3 Gengo, Morioka, Obu, Aichi 474-8522, Japan; 2 Department of Vascular Dementia, National Institute for Longevity Sciences, National Center for Geriatrics and Gerontology, 36-3 Gengo, Morioka, Obu, Aichi 474-8522, Japan; 3 Department of Bioscience, Integrated Center for Science (INCS), Ehime University, Shizukawa, Shigenobu-cyo, Onsengun, Ehime 791-0295, Japan

## Abstract

**Background:**

Apolipoprotein E allele ε4 (apoE4) is a strong risk factor for developing Alzheimer's disease (AD). Secreted apoE has a critical function in redistributing lipids among central nervous system cells to maintain normal lipid homeostasis. In addition, previous reports have shown that apoE4 is cleaved by a protease in neurons to generate apoE4(1–272) fragment, which is associated with neurofibrillary tanglelike structures and mitochondria, causing mitochondrial dysfunction. However, it still remains unclear how the apoE fragment associates with mitochondria and induces mitochondrial dysfunction.

**Results:**

To clarify the molecular mechanism, we carried out experiments to identify intracellular apoE-binding molecules and their functions in modulating mitochondria function. Here, we found that apoE4 binds to ubiquinol cytochrome *c *reductase core protein 2 (UQCRC2) and cytochrome C1, both of which are components of mitochondrial respiratory complex III, and cytochrome *c *oxidase subunit 4 isoform 1 (COX IV 1), which is a component of complex IV, in Neuro-2a cells. Interestingly, these proteins associated with apoE4(1–272) more strongly than intact apoE4(1–299). Further analysis showed that in Neuro-2a cells expressing apoE4(1–272), the enzymatic activities of mitochondrial respiratory complexes III and IV were significantly lower than those in Neuro-2a cells expressing apoE4(1–299).

**Conclusion:**

ApoE4(1–272) fragment expressed in Neuro2a cells is associated with mitochondrial proteins, UQCRC2 and cytochrome C1, which are component of respiratory complex III, and with COX IV 1, which is a member of complex IV. Overexpression of apoE4(1–272) fragment impairs activities of complex III and IV. These results suggest that the C-terminal-truncated fragment of apoE4 binds to mitochondrial complexes and affects their activities, and thereby leading to neurodegeneration.

## Background

It has been shown that the prevalence of Alzheimer's disease (AD) is associated with the polymorphisms of genes related to cholesterol metabolism, including *apolipoprotein E (apoE) *[1-3], *ATP-binding cassette transporter A1 (ABCA1) *[4], and *CYP46*, the gene encoding cholesterol 24-hydroxylase [5,6]. Human apoE, a 34-kDa protein with 299 amino acids, has three major isoforms, apoE2, apoE3, and apoE4 [7-9]. It is well known that the possession of apoE4 allele is a major risk factor for Alzheimer's disease (AD) [1-3]. The apoE4 allele, which is found in 40–65% of cases of sporadic and familial AD, increases the occurrence and lowers the age of onset of the disease [3,10]. In the central nervous system, apoE is one of the major lipid acceptors [11,12] and interacts with ABCA1 [13] to remove cholesterol from cells and generate high-density lipoprotein (HDL) particles [14] in an apoE-isoform-specific manner [15-18]. Because apoE-HDL is the major cholesterol supplier in the brain and the supply of HDL-cholesterol is essential for synaptogenesis and neurite outgrowth in neurons [19,20], the apoE-isoform-dependent difference in HDL generation may result in the apoE-isoform-dependent difference in the maintenance of synaptic plasticity and the recovery of neurons from neuronal damage found in AD brains.

In addition to the role of apoE in modulating extracellular lipid transport, the isoform-dependent intracellular functions of apoE have also been reported. A previous report has shown that apoE3 recycling is associated with concomitant cholesterol efflux and thereby contributes to the formation of apoE-containing HDL, whereas apoE4 recycling is impaired and apoE4 accumulates within endosomal compartments, inducing an impaired cholesterol efflux [21], which may lead to the accumulation of cellular cholesterol and enhanced amyloid β-protein (Aβ) generation [22]. Another effect of reduced recycling of apoE4 is due to the tight binding of apoE4 to LDLR and LRP1 in the endosomal compartment [21], which in turn affects the interaction of the amyloid precursor protein (APP) and LRP1 that is crucial for the generation of Aβ [23,24]. Other lines of evidence have shown that apoE is cleaved by a protease to generate C-terminal-truncated fragments of apoE (residues 1–272) (apoE4(1–272)) in cultured neuronal cells, and the apoE(1–272) fragment is found in the brains of AD patients and transgenic mice expressing human apoE [25,26]. This proteolytic cleavage occurs in neurons, but not in astrocytes, and C-terminal-truncated fragments of apoE accumulated in an age-dependent manner in the brains of apoE4 mice and, to a significantly lesser extent, apoE3 mice [26]. These fragments, particularly apoE4(1–272), cause AD-like neurodegeneration and memory deficits in transgenic mice expressing apoE4(1–272) [27]. These lines of evidence suggest that the intraneuronal proteolytic processing of apoE could enhance the neuropathology and promote neurodegeneration in AD brains. It has been shown that the presence of a lipid-binding region of apoE (residues 244–272) is critical for apoE fragments to exert neurotoxicity in vivo [27]. Previous studies have shown that residues 267–299 are responsible for the tetramerization of apoE in solution, and the truncation of residues 273–299 in apoE4 gives rise to the monomeric form [28], and that these hydrophobic residues appear to be responsible for the neurotoxicity caused by the C-terminal-truncated apoE4 fragments [29]. In addition to the strong neurotoxicity caused by the apoE4(1–272) fragment, it has been shown that the apoE4(1–272) fragment accumulates in filamentous neurofibrillary tanglelike structures with phosphorylated tau in the cytosol or mitochondria, inducing mitochondrial dysfunction [25-27,29]. However, it still remains unclear how the apoE fragments are transported to the filamentous cytoplasmic structures or to mitochondria, and how they associate with mitochondria and induce mitochondrial dysfunction. To address these questions, we performed experiments to identify the proteins that associate with apoE4(1–272) or intact apoE4(1–299), and determine their functions. We identified three apoE4-binding proteins, all of which are components of mitochondria. We found that these proteins preferentially bind to apoE4(1–272) than to apoE4(1–299) and that the overexpression of apoE4(1–272) fragment decreases the enzymatic activities of mitochondrial respiratory complexes III and IV in cultured cells.

## Results

### Identification of apoE4-associated proteins

**Figure 1:**
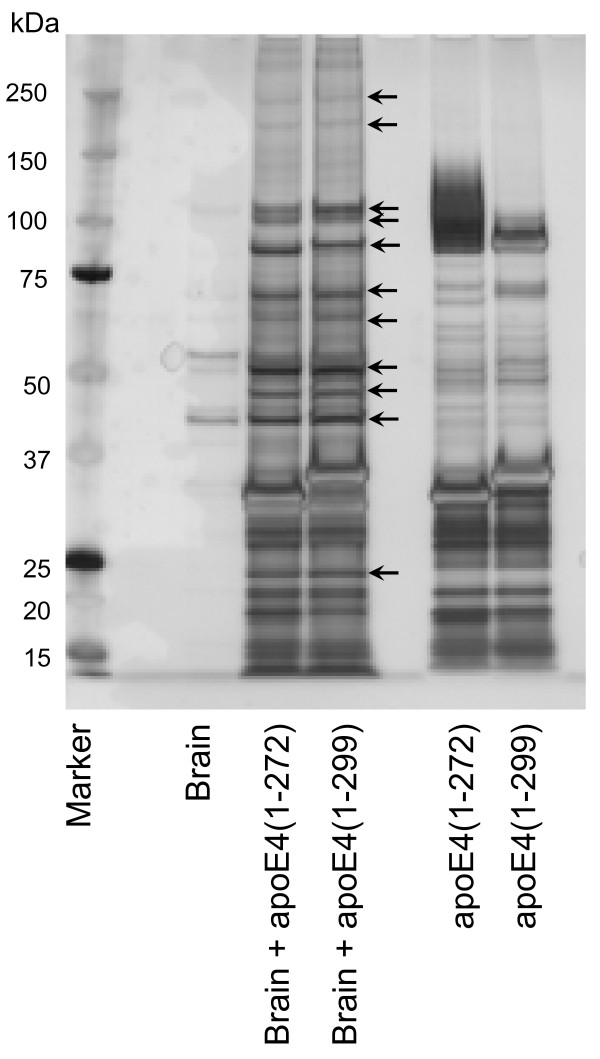
**The proteins coimmunoprecipitated with apoE4 in the membrane extracts from mouse brain**. Mouse brain membrane extracts were applied to the FLAG-apoE4(1–299) or FLAG-apoE4(1–272)-anti-FLAG M2-agarose affinity resin column and then eluted with the elution buffer. The eluant was dialyzed against the dialysis buffer, concentrated, and subjected to SDS-PAGE. The gels were stained with SilverQuest Silver Staining kit (Invitrogen). The protein bands (arrows), which were absent in the brain samples without apoE4s, and in the apoE4s samples without brain, were subjected to LC-MS/MS analysis.

To identify molecules that specifically bind to apoE4, various fractions obtained from mouse brain were loaded onto a FLAG-apoE4(1–272) or FLAG-apoE4(1–299) affinity column. The proteins bound to these columns were eluted with an excess amount of FLAG peptide and the extracts were subjected to SDS-PAGE and silver staining. When each mouse brain fraction was loaded onto a FLAG-apoE4(1–272) or FLAG-apoE4(1–299) affinity column and eluted samples were subjected to SDS-PAGE, many protein bands were detected as candidate apoE4-associated proteins (Fig. 1). These bands were detected only when the mouse brain fractions and FLAG-apoE4 recombinant proteins coexisted.

To identify these proteins, the protein bands were subjected to LC-MS/MS analysis. As a result, we identified sixteen apoE4-associated proteins, which are shown in Table 1. These candidate proteins include the ATP synthase protein α and β subunits, which were previously reported [30]. Interestingly, ten among the candidate proteins are shown to be associated with mitochondria.

**Table 1:** ApoE-associated proteins identified by LC-MS/MS analysis

Protein	Intracellular localization	Function
Solute carrier family 25 (mitochondrial carrier, Aralar) member 12	Mitochondria	Calcium-dependent mitochondrial aspartate and glutamate carrier

Ubiquinol cytochrome c reductase core protein 1	Mitochondria	Mitochondrial electron transport

* Ubiquinol cytochrome c reductase core protein 2 (UQCRC2)	Mitochondria	Mitochondrial electron transport

* Cytochrome C1	Mitochondria	Mitochondrial electron transport

Cytochrome oxidase subunit II	Mitochondria	Mitochondrial electron transport

* Cytochrome c oxidase subunit IV isoform 1 (COX IV1)	Mitochondria	Mitochondrial electron transport

ATP synthase, H+ transporting, mitochondrial F1 complex, α subunit, isoform 1	Mitochondria	ATP synthesis

ATP synthase, H+ transporting, mitochondrial F1 complex, β subunit	Mitochondria	ATP synthesis

ATP synthase, H+ transporting, mitochondrial F1 complex, δ subunit	Mitochondria	ATP synthesis

Methylenetetrahydrofolate dehydrogenase (NADP+ dependent)- 1 like	Mitochondria	Folic acid and derivative biosynthetic process

Syntaxin binding protein 1	Cytoplasm	Modulates exocytosis of dense-core granules

Nonmuscle myosin heavy chain	Cytoplasm	Actin filament-based movement

Tubulin, alpha 1A	Cytoplasm	Constituent of microtubules

RAB3A, member RAS oncogene family	Cytoplasm	Involved in exocytosis by regulating a late step in synaptic vesicle fusion.

Progesterone receptor membrane component 1	Plasma membrane	Receptor for progesterone

Cardiotrophin-like cytokine factor 1	Extracellular space	Cell surface receptor linked signal transduction

ApoE4-associated protein bands that were detected with SDS-PAGE were prepared and analyzed as described in the Methods. Asterisks (*) show the apoE-binding proteins identified and characterized in this study.

### Association of identified proteins with apoE4 in cultured cells

Next, we examined whether these proteins really associate with apoE in living cells. We cotransfected each candidate protein and FLAG-apoE4 into Neuro2a cells. Twenty-four hours following the transfection, the cells were harvested and treated with Triton X-100 solubilization buffer to obtain cell lysate as described in Materials and Methods. We carried out immunoprecipitation using these samples with an anti-FLAG antibody. The immunoprecipitate was then subjected to western blotting analysis using antibody specific for each protein or anti-HA antibody. This is because, in case we could not find antibodies specific for some candidate proteins, we could still generate HA-tagged proteins. Among the proteins identified by LC-MS/MS analysis, we found that three proteins, ubiquinol cytochrome c reductase core protein 2 (UQCRC2), cytochrome C1, and cytochrome *c *oxidase subunit 4 isoform 1 (COX IV 1) were associated with apoE4(1–299) and apoE4(1–272).

**Figure 2:**
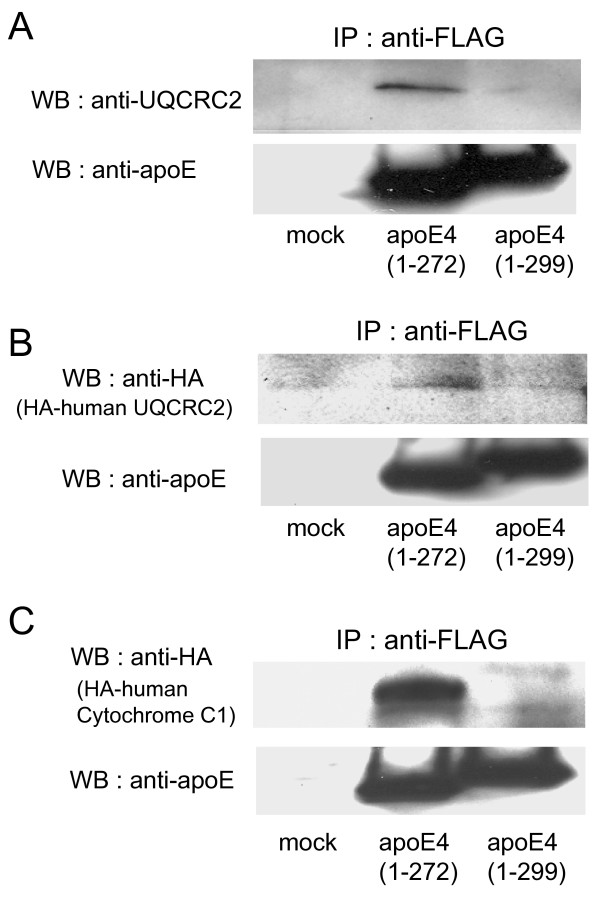
**ApoE4 interacts with the subunits of mitochondrial respiratory complex III in Neuro2a cells**. Neuro2a cells were cotransfected with mammalian expression plasmids encoding FLAG-apoE4(1–272) or FLAG-apoE4(1–299) and plasmids encoding mouse UQCRC2, human HA-UQCRC2, or human HA-cytochrome C1, all of which are candidate proteins suggested to be associated with apoE4 (Table 1). Twenty-four hours following the transfection, the cells were harvested and treated with 500 μl of Triton X-100 solubilization buffer to obtain cell lysate. The cell lysate was then incubated with anti-FLAG M2-agarose affinity resin, and the protein binding to the affinity column was eluted using FLAG peptide, and the eluted protein was then analyzed by western blotting with anti-UQCRC2 (mouse UQCRC2) antibody (A), anti-HA antibody (human UQCRC2) (B), or anti-HA antibody (human cytochrome C1) (C).

To examine whether mouse UQCRC2 is really associated with apoE proteins in cells, Neuro2a cells were cotransfected with mammalian expression plasmids encoding FLAG-apoE4(1–272) or FLAG-apoE4(1–299) and plasmids encoding mouse UQCRC2. Western blot analysis showed that the signal representing mouse UQCRC2 was clearly detected in the immunoprecipitate from the cells cotransfected with apoE4(1–272), while a very faint signal for UQCRC2 was detected in that from the cells cotransfected with apoE4(1–299). ApoE proteins were similarly immunoprecipitated in both samples (Fig. 2A). These results suggest that UQCRC2 prefers to associate with apoE4(1–272) than with apoE4(1–299).

To examine whether human UQCRC2 also associates with apoE4, we performed an experiment using human HA-tagged UQCRC2 expression vector. Similar to the results using mouse UQCRC2-transfected cells, the signals representing human HA-tagged UQCRC2 were clearly detected in the immunoprecipitate from the cells cotransfected with apoE4(1–272), while a very faint signal for HA-UQCRC2 was detected in that from the cells cotransfected with apoE4(1–299). Under these conditions, apoE4(1–272) and apoE4(1–299) proteins were similarly immunoprecipitated in both samples (Fig. 2B).

Next, we examined the association of human cytochrome C1 and apoE proteins using Neuro2a cells cotransfected with HA-tagged human cytochrome C1 expression vector and plasmids encoding FLAG-apoE4(1–272) or FLAG-apoE4(1–299). A strong signal for HA-tagged human cytochrome C1 was detected in the immunoprecipitate from the cell samples expressing apoE4(1–272), while a very faint signal for cytochrome C1 was detected in those transfected with apoE4(1–299) plasmid. ApoE4(1–272) and apoE4(1–299) proteins were similarly detected in both samples (Fig. 2C).

**Figure 3:**
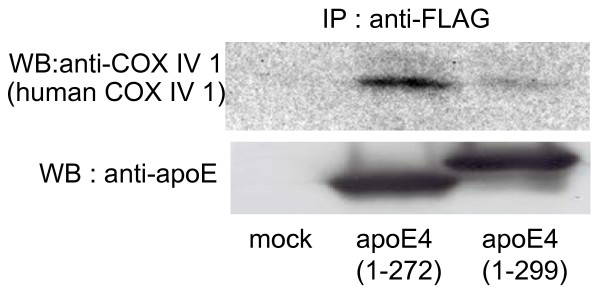
**ApoE4 interacts with the subunits of mitochondrial respiratory complex IV in Neuro2a cells**. Neuro2a cells were co-transfected with FLAG-apoE4 (1–272 or 1–299) plasmids and mammalian expression plasmids encoding the candidate apoE4-associated proteins. The cells were treated with 500 μl of Triton X-100 solubilization buffer and the cell lysate was incubated with anti-FLAG M2-agarose affinity resin. The immunoprecipitates were then analyzed by western blotting with an anti-COX IV 1 antibody (human COX IV 1).

In addition, whether COX IV 1 is associated with apoE was determined. Human COX IV 1 was immunoprecipitated in the samples from apoE4(1–272)-expressing cells and a very faint signal for COX IV 1 was detected in the samples from apoE4(1–299)-expressing cells, whereas apoE4(1–272) and apoE4(1–299) proteins were similarly immunoprecipitated in both samples (Fig. 3). Interestingly, these proteins, UQCRC2, cytochrome C1, and COX IV 1, are associated more strongly with apoE4(1–272) than with apoE4(1–299). Concerning other proteins, we carried out similar experiments; however, no association of these proteins with apoE4(1–272) and apoE4(1–299) was found in cultured cells (data not shown).

### The levels of apoE4 in mitochondrion-rich fraction isolated from ApoE4(1–272)- or ApoE4(1–299)-expressing cells

**Figure 4:**
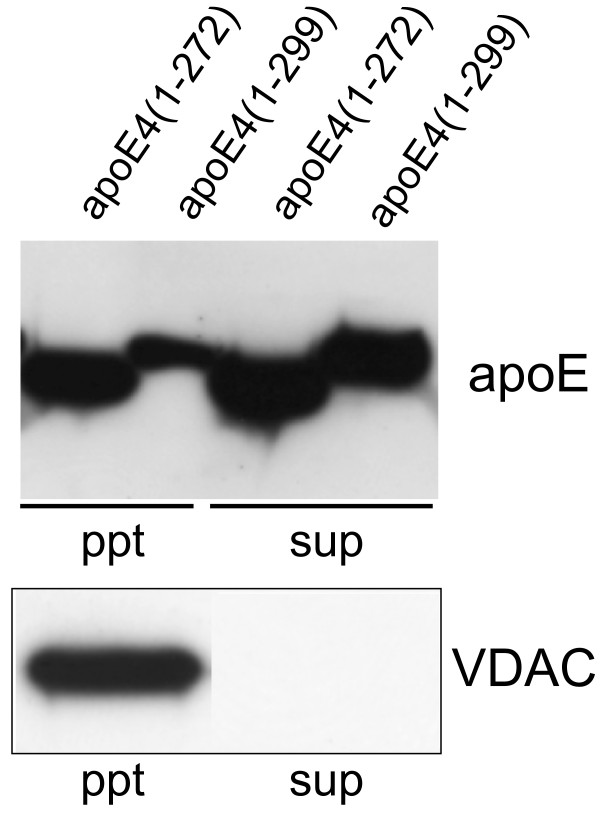
**The level of apoE4(1–272) recovered from the mitochondrion-rich fraction is greater than that of apoE4(1–299)**. Neuro2a cells transfected with ApoE4(1–272) and ApoE4(1–299) plasmids were harvested and homogenized with a homogenizing buffer (10 mM Tris-HCl, pH 7.4, 1 mM EDTA, 0.25 M sucrose), and the resulting homogenate was centrifuged at 1,000 *g *for 10 min at 4°C. The resulting supernatant was further centrifuged at 8,000 *g *for 20 min at 4°C. The resulting precipitate (ppt) was used as the mitochondrion-rich fraction. Equal amounts of proteins from the ppt and supernatant (sup) fractions were analyzed by western blot analysis using the anti-apoE antibody, AB946, and the anti-VDAC antibody. VDAC was used as the mitochondrion marker.

The results indicate that apoE4(1–272) binds to mitochondrial proteins; therefore, we next determined whether the levels of apoE4(1–272) and apoE4(1–299) are also associated with the mitochondria. We, thus, determined the levels of apoE4(1–272) and apoE4(1–299) in mitochondrion-rich fractions isolated from Neuro2a cells transfected with apoE4(1–272) or apoE4(1–299). The level of apoE4(1–272) in the pellet, a mitochondrion-rich fraction, was greater than that of apoE4(1–299) (Fig. 4). VDAC, a mitochondrion marker, was recovered in the pellet fraction (Fig. 4).

### Effect of apoE4(1–272) overexpression on activities of mitochondrial respiratory complexes

**Figure 5:**
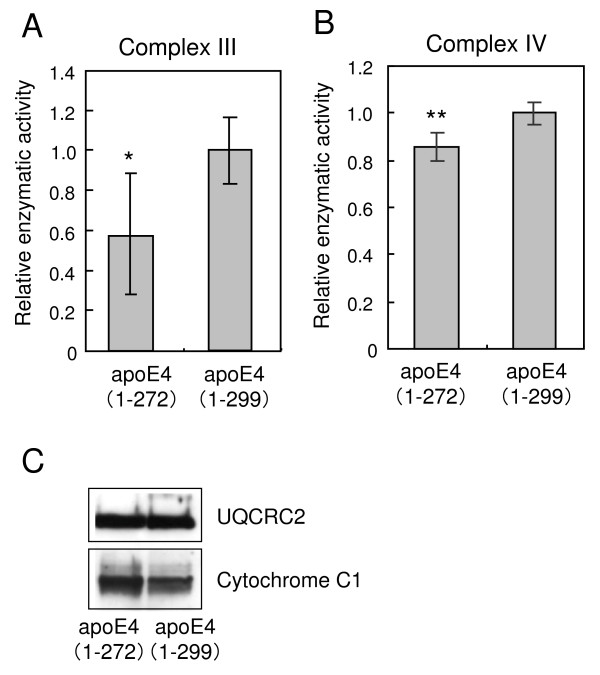
**Overexpression of apoE4(1–272) results in the decreased level of complex III and IV activities**. Enzymatic assays of respiratory chain complexes III (A) and IV (B) from Neuro2a cells overexpressing FLAG-apoE4(1–272 or 1–299) were determined as described in the Methods. The mitochondria levels in apoE4(1–272)- and apoE4(1–299)-overexpressing cells were determined by western blot analysis using the anti-UQCRC2 and the anti-cytochrome C1 antibodies (C). Data are the mean ± SEM of nine independent experiments. * *P *< 0.005, ** *P *< 0.0005 (t-distribution test).

It is known that UQCRC2 and cytochrome C1 are subunits of mitochondrial respiratory complex III and COX IV 1 is a subunit of mitochondrial respiratory complex IV. It was reported that apoE4(1–272) induces mitochondrial dysfunction [25-27,29]. Because these proteins are associated more strongly with apoE4(1–272) than with apoE4(1–299), we investigated whether the enzymatic activities of mitochondrial respiratory complexes III and IV change, when apoE4(1–272) is overexpressed in cultured cells. Complex III activity was expressed as the difference in the reduction of cytochrome *c *with or without antimycin A and myxothiazol, both of which are complex III inhibitors. Expectedly, the complex III activity of apoE4(1–272)-overexpressing Neuro2a cells was lower than that of apoE4(1–299)-overexpressing cells (Fig. 5A). Because apoE4(1–272) associates with UQCRC2 and cytochrome C1, there was a possibility that the decrease in complex III activity in Neuro2a cells expressing apoE4(1–272) was due to the interaction between apoE4 and complex III. Complex IV activity was expressed as the difference in the oxidation of ferrocytochrome C with or without KCN and Na_3_N, both of which are complex IV inhibitors. The complex IV activity of apoE4(1–272)-overexpressing cells was significantly lower than that of apoE4(1–299)-overexpressing cells (Fig. 5B). The levels of the mitochondrial proteins UQCRC2 and cytochrome C1 in apoE4(1–272)- and apoE4(1–299)-overexpressing cells were similar, as demonstrated by western blot analysis using anti-UQCRC2 and anti-cytochrome C1 antibodies (Fig. 5C).

### Effects of overexpression of apoE4(1–272) and apoE4(1–299) on ATP synthase activity and mitochondrial membrane potential

**Figure 6:**
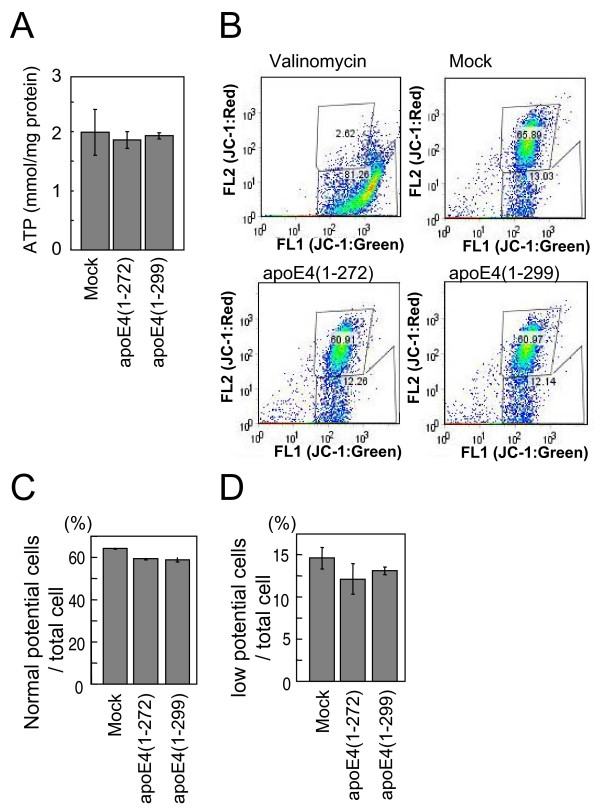
**Effects of overexpression of apoE4(1–272) and apoE4(1–299) on ATP synthase activity and mitochondrial membrane potential**. The ATP synthase activity in Neuro2a cells transfected with the ApoE4(1–272) and ApoE4(1–299) plasmids were determined (A) as described in the Methods. The data are the mean ± SEM of three experiments. (B) Flow cytometry plots were used to determine the ratio of cells having normal and low mitochondrial membrane potentials, which were demonstrated by staining with the JC-1 dye. The distribution of the cells sorted by FACS was analyzed, and the ratios of the number of cells showing normal membrane potential (C) and low membrane potential (D) to total cell number were calculated.

Because apoE4(1–272) decreases the activities of mitochondrial complexes III and IV, we next examined whether the overexpression of apoE4(1–272) also affects ATP synthase activity and mitochondrial membrane potential. Unexpectedly, apoE4(1–272) and apoE4(1–299) showed no effect on ATP synthase activity (Fig. 6A). We further examined the effect of the overexpression of apoE4(1–272) and apoE4(1–299) on mitochondrial membrane potential. Neuro2a cells transfected with these apoE4 species were stained with JC-1, a fluorescent dye that has been shown to be a reliable indicator of mitochondrial membrane potential changes in intact cells. After three hours of treatment of Neuro2a cells with 1 μM valinomycin, a K^+ ^ionophore that disrupts the transmembrane electrical gradient, the intensity of red fluorescence (FL2) markedly decreased, whereas that of green fluorescence (FL1) slightly increased, as demonstrated by JC-1 staining (Fig. 6B), indicating the dissipation of mitochondrial membrane potential. On the other hand, there were no significant differences in FL1 and FL2 intensities among Neuro2a cells transfected with mock, apoE4(1–272) and apoE4(1–299) (Figs. 6B, C, and 6D), indicating that the overexpression of apoE4(1–272) and apoE4(1–299) has no effect on mitochondrial membrane potential.

## Discussion

Here, we show the molecules associated with apoE protein. Among the molecules identified, we show for the first time that apoE4, particularly C-terminal cleaved apoE4(1–272), binds to UQCRC2 cytochrome C1 and COX IV 1. Although apoE(1–272) has been shown to be translocated to mitochondria, it still remains unclear how the apoE fragments associate with mitochondria and induce mitochondrial dysfunction. The present study has shown that apoE4(1–272) binds to UQCRC2 cytochrome C1, a component of complex III, and COX IV 1, a component of complex IV, and that overexpression of apoE(1–272) fragment in Neuro2a cells results in decreases in the levels of complex III and complex IV activities compared with those in cells overexpressing intact apoE4. These results suggest that the apoE4(1–272) fragment binds to UQCRC2 cytochrome C1 and COX IV 1, thereby inhibiting complex III and complex IV activities, respectively. The candidate molecules, which may associate with apoE to transport apoE to mitochondria, were not identified in our present analysis (Table 1). This should be addressed in a future study.

Another finding in the present study is that UQCRC2 cytochrome C1 and COX IV 1 are associated more strongly with ApoE4(1–272) than with intact apoE4(1–299) (Figs. 2, 3). There are at least two possible explanations for this result. One explanation is that apoE4(1–272) is structurally different from apoE4(1–299), resulting in the difference in hydrophobicity or binding affinity to other proteins. It has been shown that the C-terminus of apoE (residues 253–289) participates in hydrophobic interactions that stabilize the tetramer [28], and that these hydrophobic residues are suggested to be responsible for inducing neurotoxicity caused by the C-terminal-truncated apoE4 [29]. In addition, a recent study has shown that apoE4 lacking a hydrophobic C-terminal α-helical segment (residues 273–299) found in brain leads to a less organized C-terminal structure that is available for interaction with cell membranes and other proteins such as Aβ [31]. Another explanation is that more apoE(1–272) is translocated to mitochondria than intact apoE(1–299), because the silver staining shows that the intensities of the bands representing apoE-associated proteins were not different between the samples containing apoE4(1–272) and apoE4(1–299) (Fig. 1), whereas the level of apoE4(1–272) was greater than that of apoE4(1–299) in the mitochondrion-rich fraction of Neuro2a cells expressing apoE4(1–272) and apoE4(1–299) (Fig. 4). Although the precise mechanism underlying this difference is yet unknown, it is possible that the truncation of residues 273–299 in apoE4 leads to the reorganization of the C-terminal domain, with a lipid-binding region being less organized and available for hydrophobic interaction including Aβ [31-33].

Regarding the mitochondrial dysfunction in Alzheimer disease, there are previous reports showing that the overexpression of amyloid precursor protein increases the level of Aβ in mitochondria [34], and that mitochondrial complex III and IV activities are decreased in Tg2576 mouse brains [34]. In addition, the complex IV activity was shown to decrease in the brain of AD patients [35-39]. Moreover, it has been shown that the apoE(1–272) fragment is generated at a greater level from apoE4 than apoE3, and the overexpression of apoE isoform-dependently affects mitochondrial function [27]. These lines of evidence together with our present study suggest that the greater level of apoE4(1–272) fragment generated from apoE4 may be associated with Aβ that is transported to mitochondria and binds to UQCRC2 cytochrome C1 and COX IV 1, and causes mitochondrial dysfunction.

Complexes III and IV are related to ATP synthesis and the maintenance of mitochondrial membrane potential, which are critical for cell survival. Thus, we examined the effect of the transient expressions of apoE4(1–272) and apoE4(1–299) on ATP synthesis and membrane potential in Neuro2a cells. Unexpectedly, there was no difference between apoE4(1–272)- and apoE4(1–299)-transfected cells in terms of the levels of ATP synthesis or membrane potential (Fig. 6). However, when we tried to generate the Neuro2a cells, in which apoE4(1–272) is stably expressed, all the cells were dead within 2 weeks after the transfection, whereas apoE4(1–299)-expressing cells remained alive. These results suggest that apoE4(1–272) may have neurotoxicity as previously reported [29], although the level of toxicity is low. This issue remains to be addressed in further studies.

One may consider that apoE is synthesized as a secretory protein; however, how apoE enters the cytosol remains unclear and controversial. Previous studies have shown that apoE escapes the secretory or endosomal internalization pathway, and enters the cytosol of neuronal cells [25,29,40] and non-neuronal cells [41], whereas another study has failed to show this [42]. Therefore, the physiological relevance of the three mitochondrial proteins that we have identified in this study, which are associated with apoE, remains to be confirmed under physiological conditions.

It is well known that apoE4 is a strong risk factor for AD development, and the regulation of apoE4 function may be a therapeutic target for AD. Our findings indicate that if we could modulate the generation of apoE4(1–272) and/or modulate its translocation to mitochondria, the apoE4-associated induction of neurodegeneration could be prevented or attenuated. Because it has been shown that the C-terminus of the apoE-cleaving enzyme is a neuron-specific, chymotrypsin-like serine protease [25-27], the characterization and modulation of this enzyme activity would be a therapeutic target for AD.

## Conclusion

We identified intracellular apoE-binding molecules and determined their functions in modulating mitochondria function. The ApoE-binding molecules we found are ubiquinol cytochrome *c *reductase core protein 2 (UQCRC2), cytochrome C1, and cytochrome *c *oxidase subunit 4 isoform 1 (COX IV 1). The UQCRC2 and cytochrome C1 are components of mitochondrial respiratory complex III, and COX IV 1 is a component of complex IV. Interestingly, these proteins associated with apoE4(1–272) more strongly than intact apoE4 (1–299). When apoE4(1–272) expression level increased in Neuro2a cells, the enzymatic activities of mitochondrial respiratory complexes III and IV were significantly lower than those in Neuro-2a cells expressing apoE4(1–299). These results suggest that the C-terminal-truncated fragment of apoE4(1–272) bind to mitochondrial complexes and affects their activities.

## Methods

### Preparation of mouse brain membrane extracts and cytosolic fraction

Brains obtained from C57BL6 male mice were homogenized with a homogenizing buffer (10 mM Tris-HCl, pH 7.4 1 mM EDTA 0.25 M sucrose), and the homogenate was centrifuged at 1,000 *g *for 10 min at 4°C. The supernatant was recentrifuged at 10,000 *g *for 20 min at 4°C, and the resulting precipitate was suspended in a homogenizing buffer. The supernatant was recentrifuged at 100,000 *g *for 1 min at 4°C, and the resulting precipitate was suspended in a homogenizing buffer. The supernatant was used as the cytosolic fraction. The proteins in the 10,000 *g *and 100,000 *g *pellet fraction were extracted with homogenizing buffer containing 1 M KCl. The resulting pellet was solubilized with 2% Triton X-100 for 1 h at 4°C and then centrifuged at 100,000 *g *of 1 h at 4°C. The supernatants were used as 10,000 *g *or 100,000 *g *membrane extracts.

### FLAG fusion proteins

Recombinant FLAG-apoE4 fusion proteins encoding WT (1–299) or C-terminal truncated apoE4 (1–272) were prepared and purified as follows. PCR products encoding apoE4 (1–272) and apoE4 (1–299) were subcloned into pFLAG-MAC expression vector (Sigma). These plasmids were transformed into the BL21 strain of *Escherichia coli *and induced with isopropyl-1-thio-b-_D_-galactopyranoside to produce FLAG fusion proteins. The bacteria were suspended in PBS, and vigorous sonication was performed before centrifugation at 10,000 *g *for 20 min. The resulting supernatants were applied to anti-FLAG M2-agarose affinity resin column (Sigma) and then eluted with an elution buffer (TBS containing 100 μg/ml FLAG peptide (Sigma)). Purified FLAG fusion proteins were dialyzed against TBS.

### FLAG-ApoE4 affinity chromatography and LC-MS/MS analysis

Recombinant FLAG-apoE4(1–299) or FLAG-apoE4(1–272) fusion protein coupled to anti-FLAG M2-agarose affinity resin was used to identify affinity-purified apoE4-binding proteins. The fractionated mouse brain samples were applied to the FLAG-apoE4(1–299)- and FLAG-apoE4(1–272)-anti-FLAG M2-agarose affinity resin column. The proteins bound to the resin column were then eluted with the elution buffer. The eluted proteins were dialyzed against the dialysis buffer, concentrated, and subjected to SDS-PAGE. The gels were stained with SilverQuest Silver Staining kit (Invitrogen). The proteins specifically associated with apoEs demonstrated as silver-stained bands were cut out, digested with trypsin, and subjected to LC-MS/MS analysis.

### LC-MS/MS analysis

The proteins in the silver-stained bands were reduced with 10 mM dithiothreitol at room temperature for 2 h and alkylated with 40 mM iodoacetamide in the dark at room temperature for 30 min. Each sample was digested with trypsin (4 μg/ml; Trypsin Gold, Promega) in 40 mM NH_4_HCO_3_/10%ACN at 37°C overnight. The extracted peptides were then separated via nano liquid chromatography (LC) (Paradigm MS4, Michrom BioResources, Inc., Auburn, CA) using a Magic C18 column (0.2 × 50 mm; Michrom BioResources, Inc.; Auburn, CA). The LC eluent was analyzed using an LCQ Advantage MAX mass spectrometer (Thermo Fisher Scientific) equipped with an ion-spray source. All MS/MS spectra were searched by SEQUEST algorithm from BioWorks software (Thermo Fisher Scientific).

### Cells

Neuro2a cells were grown in DMEM medium, supplemented with 10% FBS, 50 units/ml penicillin, 50 mg/ml streptomycin, and 2 mM glutamine at 37°C in a humidified 5% CO_2 _95% air incubator.

### Transfection of plasmids into cells and co-immunopricipitation

PCR products encoding apoE4 (1–272) and apoE4 (1–299) were subcloned into pFLAG-CMV-2 expression vector (Sigma). Neuro2a cells were co-transfected with FLAG-apoE4 (1–272 or 1–299) plasmids and mammalian expression plasmids coding the candidate of apoE4 associating proteins (Toyobo, Japan) using Lipofectamine 2000 (Invitrogen, CA, USA). As necessary, mammalian expression plasmids coding the candidate apoE4-associated proteins were fused with HA-tag of their C-terminus using KOD-PLUS Mutagenesis Kit (Toyobo). The plasmids were transfected to Neuro2a cells as follows. Neuro2a cells were plated on a 6-cm plate at a cell density of 1.5 × 10^6 ^and cultured in the culture medium described above. The next day, the ells were transfected with the plasmid employing Lipofectamine 2000 reagent. On culture day 3, the cells were lysed with 500 μl of Triton X-100 solubilization buffer (10 mM Tris-HCl (pH 7.4), 150 mM NaCl, 1 mM EDTA, 10 mg/ml leupeptin, 1 mM PMSF, 0.5% Triton X-100). The cell lysate was incubated with anti-FLAG M2-agarose affinity resin. After an overnight incubation at 4°C, the beads were washed three times with Triton X-100 solubilization buffer and then eluted with the elution buffer (PBS containing FLAG peptide at a concentration of 100 μg/ml). The immunoprecipitates were then analyzed by western blotting with anti-HA monoclonal antibody (Sigma), anti-UQCRC2 monoclonal antibody (Abcam), and anti-COX IV 1 monoclonal antibody (Cell Signaling).

### Enzymatic analysis of complexes III and IV

Mitochondria isolated from homogenates of Neuro2a cells transfected with ApoE4 (1–299 or 1–272) were used for enzymatic analysis.

#### (a) Complex III (Ubiquinol cytochrome c reductase)

The oxidation of ubiquinol_2 _by complex III was determined using cytochrome *c *(III) as an electron acceptor. The assay was carried out in an assay medium (25 mM potassium phosphate buffer (pH 8.0), 1 mM EDTA, 1 mM KCN, 3 mM Na_3_N) supplemented with 20 μM cytochrome *c *(III), and 20 μM ubiquinol_2_. The reaction was started with 5 μg of mitochondrial protein and the enzyme activity was measured at 550 nm. The activity of complex III is estimated to be the difference in the reduction of cytochrome *c *with and without 10 μg/ml antimycin A and 10 μg/ml myxothiazol.

#### (b) Complex IV (Cytochrome c Oxidase)

The enzyme activity of cytochrome *c *oxidase (complex IV) was determined using Mitochondria Activity Assay kit (BioChain Institute, U.S.A.) and performed following the manufacture's procedure. Complex IV activity was measured as the oxidation of ferrocytochrome *c *by cytochrome *c *oxidase at 550 nm. Complex IV activity is expressed as the difference in the oxidation of ferrocytochrome C with or without KCN and Na_3_N as complex IV inhibitor.

### ATP synthase activity assay

Neuro2a cells transfected with apoE4(1–272) and apoE4(1–299) plasmids were harvested and homogenized with a homogenizing buffer (10 mM Tris-HCl, pH 7.4, 1 mM EDTA, 0.25 M sucrose), and the homogenate was centrifuged at 1,000 *g *for 10 min at 4°C. The resulting supernatant was further centrifuged at 10,000 × g for 20 min at 4°C, and the resulting pellet (ppt) fractions were obtained. The ppt fractions were resuspended in 100 μl of assay buffer (1 mM ADP and 5 mM sodium succinate) and incubated for 5 min at 37°C. 100 mM Tris-HCl buffer (pH 7.6) containing 4 mM EDTA was added and the fractions were further incubated for 2 min at 100°C. Then the fractions were plated on ice and ATP level in each fraction was determined using an ATP bioluminescence assay kit CLS II (Roche Diagnosis GmbH, Mannheim, Germany).

### Analysis of mitochondrial membrane potential (mtΔϕ)

ApoE4(1–272 or 1–299)-transfected Neuro2a cells were stained with 2 μM JC-1 (Molecular Probes, Eugene, OR) at 37°C for 20 min. Then, the cells were analyzed using a flow cytometer FACSCalibur (Becton Dickinson, Franklin Lakes, NJ) with FlowJo software (Tree Star Inc., Ashland, OR).

### Statistical analysis

StatView computer software (Windows) was used for statistical analysis. Statistical significance of differences between samples was evaluated by multiple pairwise comparisons among the sets of data using ANOVA and the Bonferoni t-test.

## Competing interests

The authors declare that they have no competing interests.

## Authors' contributions

TN carried out major part of the experiments. AW carried out TOF-MS/MS analysis and identified molecules associated with apoE. TF generated plasmid containing intact apoE3 and apoE4 cDNA. TH prepared cultured cells. MM designed this study and was involved in the interpretation of the results and in drafting the manuscript.
